# Perioperative patient-specific factors-based nomograms predict short-term periprosthetic bone loss after total hip arthroplasty

**DOI:** 10.1186/s13018-020-02034-5

**Published:** 2020-11-02

**Authors:** Guangtao Fu, Mengyuan Li, Yunlian Xue, Qingtian Li, Zhantao Deng, Yuanchen Ma, Qiujian Zheng

**Affiliations:** 1 Division of Orthopedics, Guangdong Provincial People’s Hospital, Guangdong Academy of Medical Sciences, Guangzhou, Guangdong Province People’s Republic of China; 2 Division of Statistics, Guangdong Provincial People’s Hospital, Guangdong Academy of Medical Sciences, Guangzhou, Guangdong Province People’s Republic of China

**Keywords:** Prediction tool, Nomogram, Periprosthetic bone loss, Total hip arthroplasty, Gruen zones

## Abstract

**Background:**

Although medical intervention of periprosthetic bone loss in the immediate postoperative period was recommended, not all the patients experienced periprosthetic bone loss after total hip arthroplasty (THA). Prediction tools that enrolled all potential risk factors to calculate an individualized prediction of postoperative periprosthetic bone loss were strongly needed for clinical decision-making.

**Methods:**

Data of the patients who underwent primary unilateral cementless THA between April 2015 and October 2017 in our center were retrospectively collected. Candidate variables included demographic data and bone mineral density (BMD) in spine, hip, and periprosthetic regions that measured 1 week after THA. Outcomes of interest included the risk of postoperative periprosthetic bone loss in Gruen zone 1, 7, and total zones in the 1st postoperative year. Nomograms were presented based on multiple logistic regressions via R language. One thousand Bootstraps were used for internal validation.

**Results:**

Five hundred sixty-three patients met the inclusion criteria were enrolled, and the final analysis was performed in 427 patients (195 male and 232 female) after the exclusion. The mean BMD of Gruen zone 1, 7, and total were decreased by 4.1%, 6.4%, and 1.7% at the 1st year after THA, respectively. 61.1% of the patients (261/427) experienced bone loss in Gruen zone 1 at the 1st postoperative year, while there were 58.1% (248/427) in Gruen zone 7 and 63.0% (269/427) in Gruen zone total. Bias-corrected C-index for risk of postoperative bone loss in Gruen zone 1, 7, and total zones in the 1^st^ postoperative year were 0.700, 0.785, and 0.696, respectively. The most highly influential factors for the postoperative periprosthetic bone loss were primary diagnosis and BMD in the corresponding Gruen zones at the baseline.

**Conclusions:**

To the best of our knowledge, our study represented the first time to use the nomograms in estimating the risk of postoperative periprosthetic bone loss with adequate predictive discrimination and calibration. Those predictive models would help surgeons to identify high-risk patients who may benefit from anti-bone-resorptive treatment in the early postoperative period effectively. It is also beneficial for patients, as they can choose the treatment options based on a reasonable expectation following surgery.

## Introduction

Total hip arthroplasty (THA) is the most effective therapy for end-stage hip diseases. Over 500,000 THAs were performed in the USA annually, and the demand for THA is still growing [1]. As one of the major concerns after THA, periprosthetic bone loss was closely related to aseptic loosening, periprosthetic fractures, and implant failure [2]. It was reported that the mean periprosthetic bone loss was up to 21.9% 10 years post-operation [3, 4]. Thus, medical intervention of periprosthetic bone loss in the immediate postoperative period was widely accepted [5, 6]. However, not all the patients experienced periprosthetic bone loss after THA [7, 8]. Correspondingly, identification of patients with increased risk of postoperative periprosthetic bone loss is of great value for surgeons when making clinical decisions and cost-effect analysis.

Many patient-specific and surgery-related factors were closely related with increased risk of postoperative prosthetic bone loss, including age [9], body mass index (BMI) [10], primary diagnosis [11], femoral stem design [12], preoperative bone mineral density (BMD) in hip and spine [13], periprosthetic BMD measured in the immediate postoperative period [14], and the administration of anti-osteoporosis agents [5]. Knowledge of these variables, however, only provides the surgeons with an individual factor that improves or worsens specific outcome. No study yet has provided a comprehensive tool that enables a quantified individualized risk prediction of postoperative periprosthetic bone loss on bias of numerous variables. Nomogram is a pictorial representation of a complex mathematical formula designed to allow the approximate graphical computation. Its efficiency in predicting clinical outcomes after orthopedic surgery, such as 30 days readmission rate and risk of major complications, has been well demonstrated [15–17]. Thus, the purpose of the present study was to create perioperative patient-specific factors-based nomograms for postoperative periprosthetic bone loss prediction, which were applicable before the medical intervention.

## Patients and methods

The electronic medical records were retrospectively reviewed to identify patients who underwent primary unilateral cementless THA between April 2015 and October 2017 in the Center of Orthopedics, Guangdong Provincial Peoples’ Hospital. The exclusion criteria included (1) inflammatory arthritis; (2) previous history of trauma or surgery in the involved hip; (3) periprosthetic fracture or infection; (4) secondary osteoporosis or other bone metabolism disorders; (5) absence of *intact* data of periprosthetic BMD measurement. We used the TRIPOD checklist when writing our report [18].

### Data collection

Data of all the patients were retrospectively retrieved from the database of Guangdong Provincial People’s Hospital. Patient demographics, preoperative BMD of hip and spine, BMD of 7 Gruen zones measured 1 week after THA, surgical details, and preoperative bone metabolic markers were collected. Outcomes included the BMD of Gruen zone 1, 7, and total zones measured 1 year after THA.

### BMD measurement

**Fig 1:**
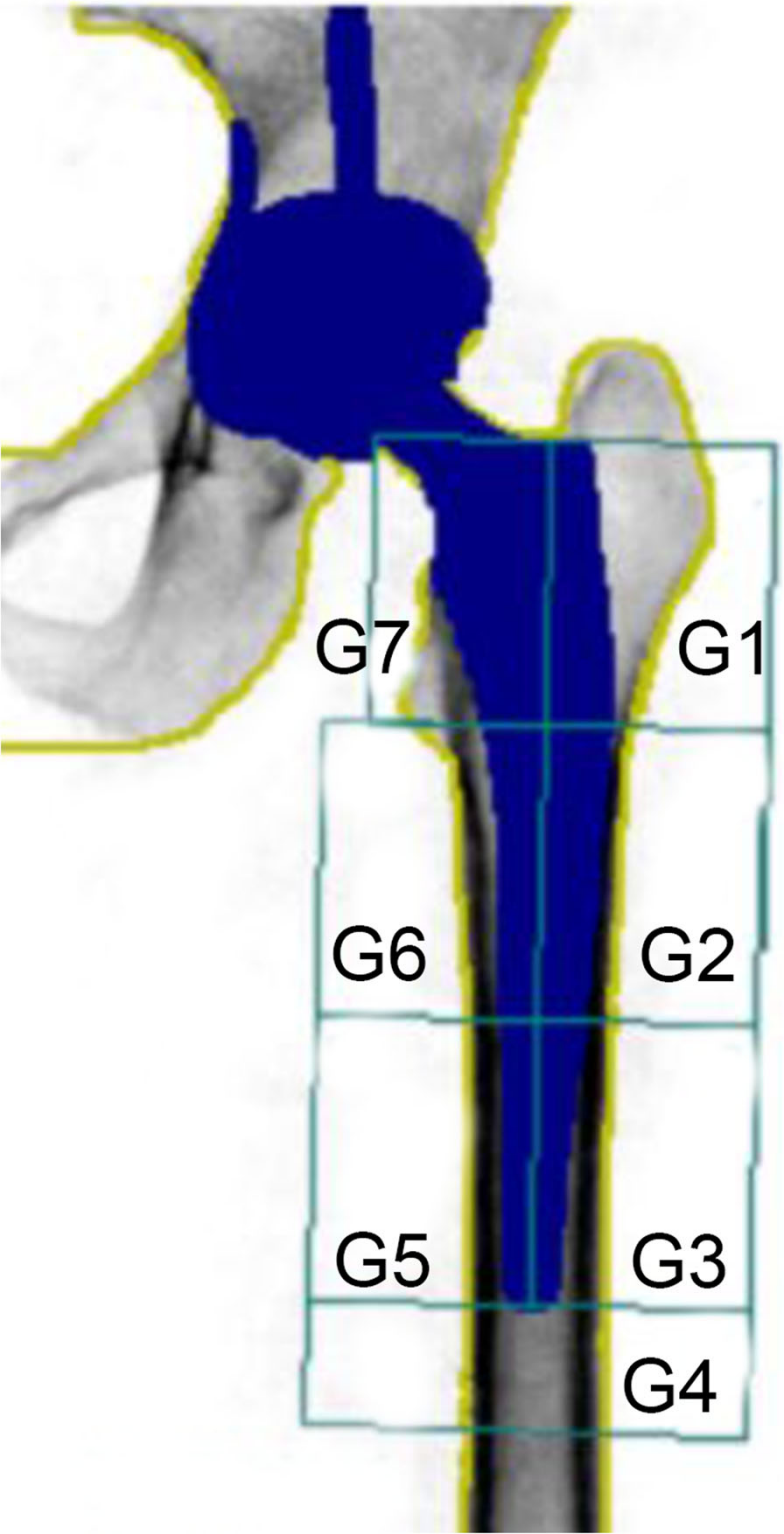
Seven Gruen zones used in dual-energy X-ray absorptiometrical analysis of postoperative periprosthetic BMD

BMD of proximal femur and lumbar spine (from L1 to L4) was measured using Dual-energy X-ray Absorptiometry (DEXA, LUNAR DPXMD#5966, Madison, WI, USA). The periprosthetic BMD of the femoral component was analyzed according to the protocol proposed by Gruen et al. [19]. Briefly, the proximolateral, lateromedial, and distolateral regions were defined as Gruen zone 1, 2, and 3, respectively. Correspondingly, the medial periprosthetic region was divided into Gruen zone 5, 6, and 7 from the proximal to distal femur. Gruen zone 4 was located at least 1 cm distal to the tip of the stem (Fig. 1). The total periprosthetic BMD was defined as the mean of BMD from zones 1 to 7. In the present study, the mean least significant changes (LSC) of the hip, spine, and periprosthetic Gruen zones were 0.017 ± 0.013 g/cm^2^, 0.007 ± 0.005 g/cm^2^, and 0.012 ± 0.015 g/cm^2^, respectively.

### Sample size

There is no golden standard approach to estimate the sample size requirements for risk prediction models till now. It was widely accepted at least 10 events per candidate variable for the derivation of a risk prediction model [20]. As 13 candidate variables were included for the regression analysis, at least 130 patients with observed positive outcomes (bone loss in Gruen zone 1, 7, or total in the 1st postoperative year) were required for the present study.

### Statistical analysis

Continuous data were expressed as mean ± standard deviation or median with interquartile range. Categorical data was present as count (percent). Prediction models for the binary outcomes were created using multivariable logistic regression. As described in the previous study, candidate variables included in the nomograms were identified in a screening step with the *P* values < 0.3 that obtained by multivariable analysis [21]. The relative importance of each predictor in the model was determined by subtracting the predictor degrees of freedom from the Wald chi-square value [16].

R version 3.5.0 (R Foundation for Statistical Computing) with specific package (rms) was utilized for the development of nomograms and all the statistical testing. For the binary outcomes, each final model achieved the maximum bias-corrected concordance index (c-index) and underwent internal validation. One thousand bootstrap samples were drawn to correct the bias, and the final model fit each sample. Predicted probabilities were obtained for the original sample based on each bootstrap estimated model and a C-index calculated. The bias-corrected C-index was defined as the average of these bootstrap c indices.

The usage of nomogram was as follows [22]. Points at respective horizontal axis represented the predictive value of the variables. After calculation of the total risk score based on the patients’ response for each variable, surgeons could correlate it to a specific chance of having the given outcome. The C index in binary outcomes predicting models represents the ability to distinguish between patients who experience an event from those who do not. It is measured on a scale of 0.5 (no better than chance) to 1 (perfect discrimination). Overall accuracy and calibration were visualized by comparing predicted versus actual probabilities, including a bias correction for overfitting.

## Results

### Descriptive data

**Fig 2:**
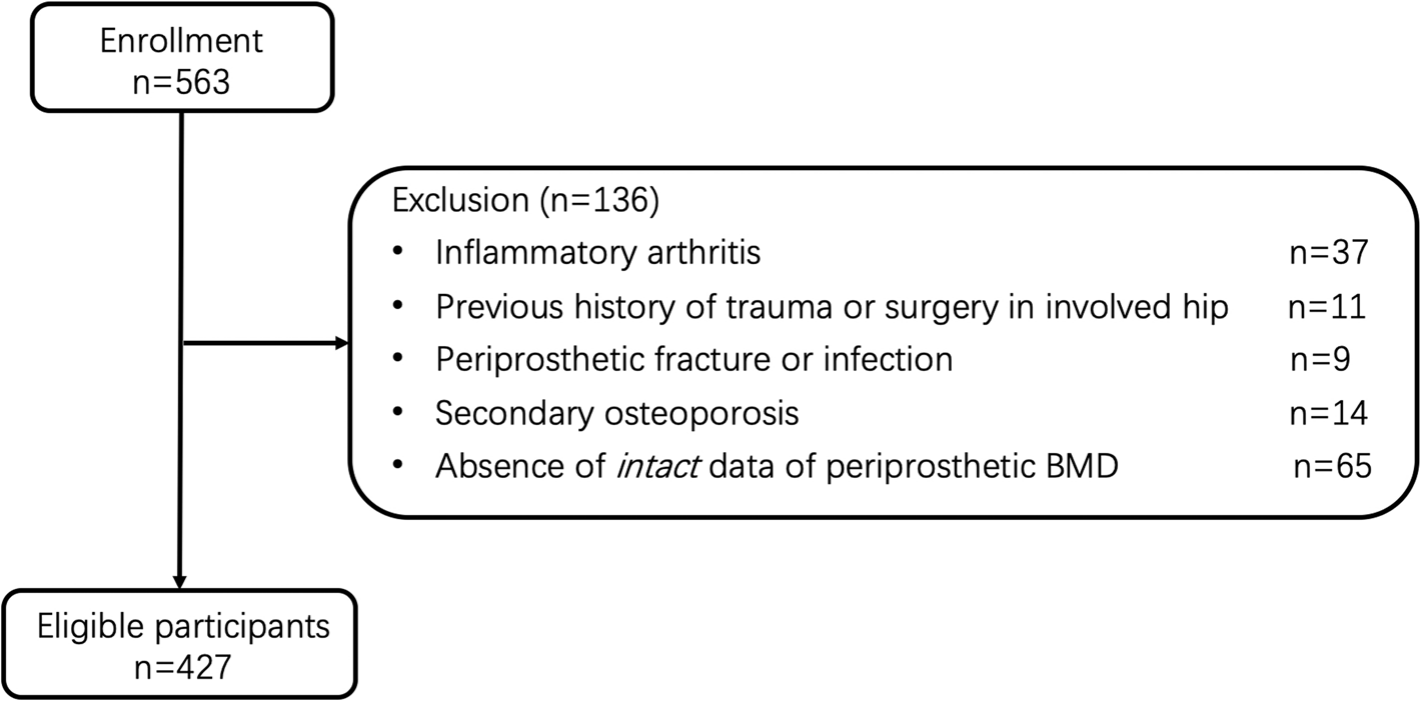
Flowchart

Five hundred sixty-three patients met the inclusion criteria were enrolled, and the final analysis was performed in 427 patients (195 male and 232 female) after the exclusion (Fig. 2). Details of the demographic data were shown in Table 1. All the surgeries were performed by the corresponding authors of the present study (QJZ and YCM). A standard posterolateral surgical approach was used. The femoral prosthesis used in the present study included the straight stem (Ribbed® classic; Waldemar Link GmbH, Germany) and the anatomic stem (L.C.U. ® classic; Waldemar Link GmbH, Germany). The CombiCup® was used for acetabular component (Waldemar Link GmbH, Germany). Partial weight bearing was required 1 week after THA, and full-weight bearing was allowed 2 weeks after THA.

**Table 1:** Demographic data

Variables	Prevalence or average
Age (years)	59.3 ± 10.8
Male	45.7% (195/427)
Married	94.1% (402/427)
BMI (kg/m^2^)	23.4 ± 3.8
Current/former smoker	39.6% (169/427)
Comorbidities	
Diabetes mellitus	17.3% (74/427)
Hypertension	36.3% (155/427)
Stroke	6.8% (29/427)
Heart dysfunction	8.7% (37/427)
Others^a^	17.1% (73/427)
Diagnosis	
Femoral neck fracture	22.7% (97/427)
Femoral head necrosis	49.6% (212/427)
Hip osteoarthritis	17.8% (76/427)
Developmental dysplasia of the hip	9.8% (42/427)
History of anti-osteoporosis therapy in the last year	20.4% (87/427)
Preoperative systematic BMD	
Spine (g/cm^2^)	0.881 ± 0.150
Hip (g/cm^2^)	0.828 ± 0.181
Serum ALP (U/L)	74.9 ± 25.6
Serum calcium (mmol/L)	2.31 ± 0.12
Preoperative Harris score of the involved hip	41.7 ± 19.9
Femoral component design	
Straight stem	65.1% (278/427)
Anatomic stem	34.9% (149/427)

^a^indicated other comorbidities including chronic obstructive pulmonary disease, pulmonary infection, dementia, Parkinson’s disease, digestive system disorders, and chronic renal failure

The mean BMD of Gruen zone 1, 7, and total was decreased by 4.1%, 6.4%, and 1.7% at the 1st year after THA, respectively (Table 2). 61.1% of the patients (261/427) experienced bone loss in Gruen zone 1 at the 1^st^ postoperative year, while there were 58.1% (248/427) in Gruen zone 7 and 63.0% (269/427) in Gruen zone total.

**Table 2:** Main outcomes

	*n*	1 week postoperative	*n*	1 year postoperative	*n*	Change (1 week–1 year postoperative)
BMD of Gruen zone 1 (g/cm^2^)	427	0.801 (0.717, 0.891)	427	0.768 (0.702, 0.842)	427	− 0.033 (− 0.116, 0.044)
BMD of Gruen zone 7 (g/cm^2^)	427	0.889 (0.716, 1.094)	427	0.807 (0.729, 0.97)	427	− 0.057 (− 0.157, 0.072)
BMD of total Gruen zones (g/cm^2^)	427	1.44 (1.323, 1.519)	427	1.395 (1.286, 1.482)	427	− 0.025 (− 0.09, 0.024)

Results were presented as median (Q1, Q3)

### Predictors for the risk of postoperative periprosthetic bone loss

**Fig. 3:**
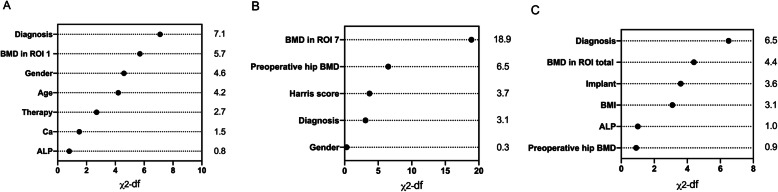
Relative importance of individual predictors within the final multivariable model for predicting bone loss in Gruen zone 1 (**a**), 7 (**b**), and total (**c**)

**Fig. 4:**
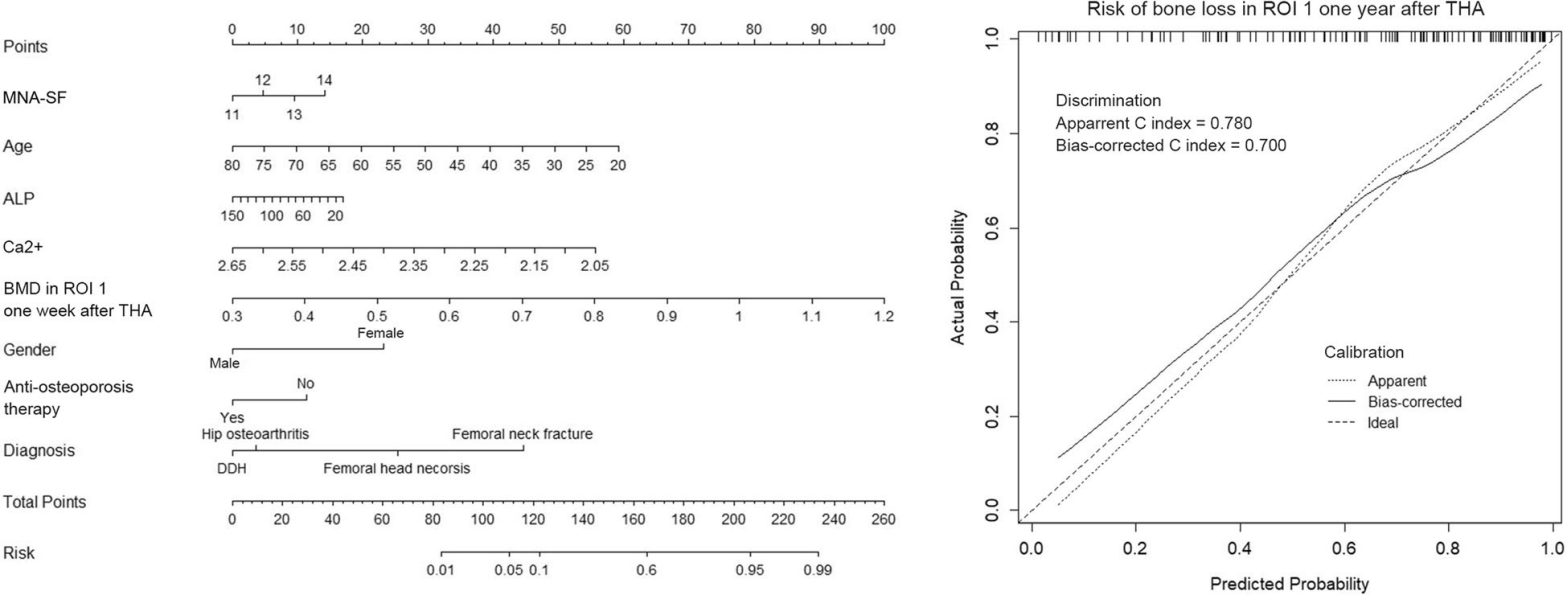
Nomogram predicting model for risk of postoperative bone loss in Gruen zone 1 and the prediction model performance

**Fig. 5:**
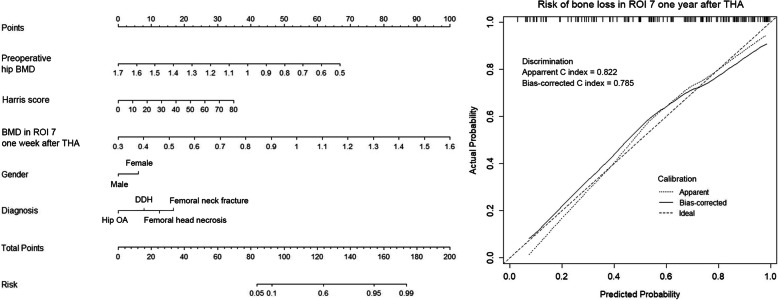
Nomogram predicting model for risk of postoperative bone loss in Gruen zone 7 and the prediction model performance measurement

**Fig. 6:**
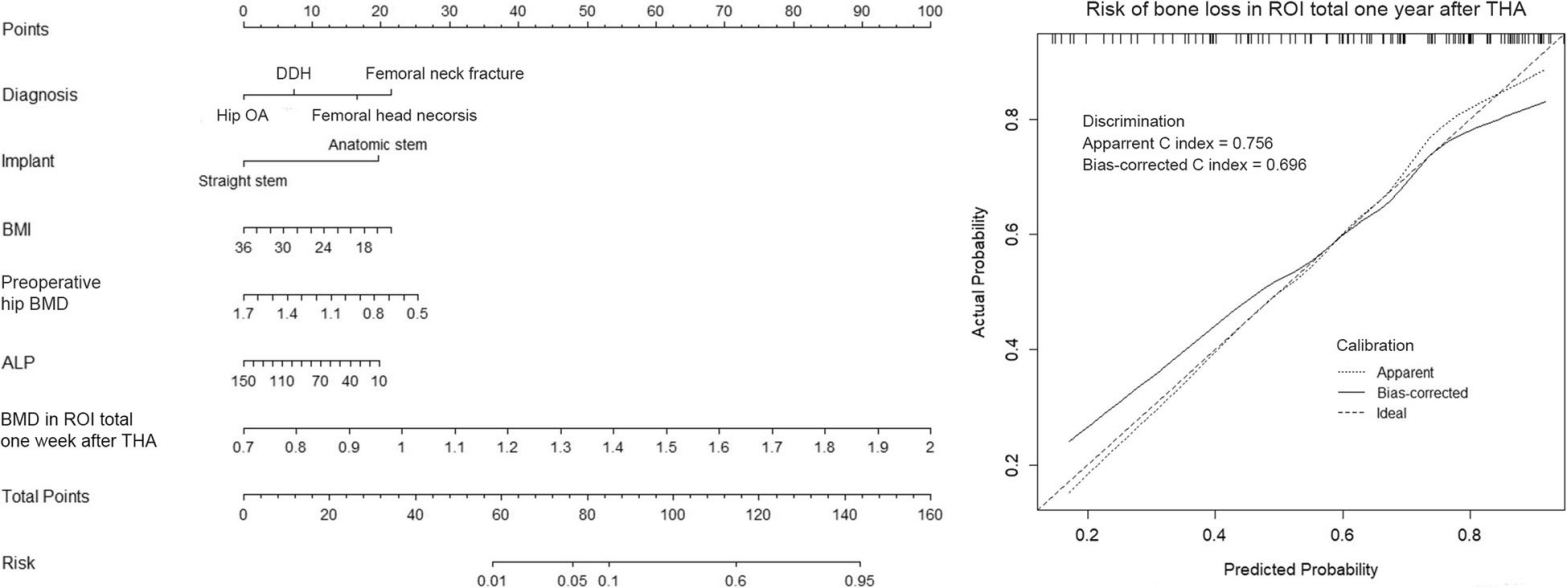
Nomogram predicting model for risk of postoperative bone loss in Gruen zone total and the prediction model performance measurement

According to the multivariable logistic regression analysis (significance *P* < 0.3), 7 variables including age, gender, diagnosis, history of anti-osteoporosis treatment, serum ALP and Ca concentration, and BMD in region of interest (ROI) 1 1 week after THA were selected to generate a predictive model via backward elimination (Table 3). The bias-corrected C-index of the entire data set was 0.700 after internal validation. For bone loss in ROI 7, the significant predictors included gender, diagnosis, preoperative hip BMD, Harris score, and BMD of Gruen zone 7 measured 1 week after THA (Table 3). In regard to ROI total, diagnosis, implant design, BMI, preoperative hip BMD, serum ALP concentration, and 1 week post-operative BMD of Gruen zone total were enrolled in the final predictive model. The bias-corrected C-index for ROI 7 and ROI total were 0.785 and 0.696, respectively. The relative predictive abilities of each selected parameter for ROI 1, 7, and total were shown in Fig. 3, while primary diagnosis and BMD in the corresponding Gruen zones at the baseline led the most values. Figures 4, 5 and 6 showed the static nomograms created from the final multivariable models of ROI 1, 7, and total.

**Table 3:** Results of the logistic regression

	ROI 1			ROI 7			ROI total		
	Coefficient	Standard error	*P* value	Coefficient	Standard error	*P* value	Coefficient	Standard error	*P* value
Age	− 0.080	0.035	0.023	0.033	0.035	0.346	− 0.020	0.027	0.454
Gender	− 1.518	0.642	0.018	− 0.856	0.763	0.262	− 0.005	0.588	0.993
History of anti-osteoporosis treatment	− 1.635	0.848	0.054	0.497	0.991	0.616	0.132	0.763	0.863
BMI	− 0.027	0.067	0.687	0.012	0.077	0.877	− 0.144	0.071	0.042
Diagnosis 1 (transform into dummy variable)	1.687	1.016	0.097	0.934	1.006	0.353	0.805	0.847	0.342
Diagnosis 2 (transform into dummy variable)	− 1.198	0.989	0.226	− 1.949	1.031	0.059	− 1.420	0.892	0.111
Diagnosis 3 (transform into dummy variable)	− 1.220	0.659	0.064	− 0.685	0.772	0.375	− 1.532	0.658	0.02
MNA-SF score	0.499	0.364	0.170	− 0.148	0.397	0.709	− 0.097	0.320	0.762
Preoperative lumbar BMD	− 2.910	2.819	0.302	2.483	3.061	0.417	0.719	2.512	0.775
Preoperative hip BMD	− 1.197	2.604	0.646	− 8.550	3.124	0.006	− 3.161	2.275	0.165
Serum ALP	− 0.017	0.013	0.180	− 0.001	0.015	0.949	− 0.018	0.012	0.154
Serum Ca	− 4.608	2.922	0.115	− 0.849	3.065	0.782	0.107	2.624	0.967
Preoperative Harris score	0.010	0.018	0.569	0.044	0.020	0.030	0.005	0.017	0.751
Implant design of femoral component	0.136	0.750	0.857	0.277	0.739	0.708	1.655	0.768	0.031
BMD of ROI 1 at 1 week after THA	7.012	2.715	0.010	/	/	/	/	/	/
BMD of ROI 7 at 1 week after THA	/	/	/	10.658	2.389	< 0.001	/	/	/
BMD of ROI total at 1 week after THA	/	/	/	/	/	/	5.705	2.461	0.020

## Discussion

Previous studies demonstrated that initial periprosthetic bone remodeling process was mainly completed in the first 12 postoperative months [4, 23]. Thus, the present study focused on 1-year periprosthetic bone loss after THA, which we believed to be most evident and clinically relevant. As majority of the femoral stems for primary THA were designed as proximally coated [24], the changes of proximal periprosthetic BMD, namely Gruen zone 1 and 7 (Fig. 1) were suggested to be more important than those of other Gruen zones. In consistent with our results, a previous study found that the decreases of the mean BMD in Gruen zone 1 and 7 varied from 5 to 10% during the first 2 years after THA [7]. As the mean changes of BMD in Gruen zone 1 (− 0.033 g/cm^2^), Gruen zone 7 (− 0.057 g/cm^2^), and total Gruen zones (− 0.025 g/cm^2^) were larger than the LSC (0.012 g/cm^2^), we believe that our results represented a real biological change [25].

As we mentioned before, not all the patients experienced periprosthetic bone loss after THA [7, 8], while early medical intervention was recommended for all the patients underwent THA [5, 6]. Numerous studies demonstrated that the administration of bisphosphonate effectively inhibited postoperative periprosthetic bone loss from 1 to 3 years after the THA [13, 26–29]. However, there is no clear guideline regarding the indication of bisphosphonate treatment for patients underwent THA, especially for those without osteoporosis and osteopenia. Traditionally, clinicians used their individual or group evaluation of the risk of postoperative periprosthetic bone loss as the basis of making clinical decisions, which has been proven to be subject to biases [21]. A prediction model that allows estimation of postoperative periprosthetic BMD changes at perioperative period could enable efficient identification of patients who benefit more from bisphosphonate treatment and individualized decision-making. Such prediction model could also provide patients with reasonable expectations following surgery, which may improve satisfaction and patient compliance. However, no predictive tool that enables simplified, quantified individualized risk evaluations of postoperative periprosthetic bone loss on bias of numerous variables was available till now.

Nomograms have been widely used in predicting clinical outcomes after orthopedic surgery [15–17, 30]. Those prediction models that individualized the predicted outcome to specific patients’ characteristics performed better than simply relying on the average outcome [21]. To the best of our knowledge, our study represented the first time to use the nomograms in estimating the risk of postoperative periprosthetic bone loss. In the present study, variables (age, BMI, implant design, etc) that have been reported to be potential risk factors of postoperative periprosthetic BMD decreases were retrospectively collected to create the nomograms [9–14]. As the bias-corrected C index of Gruen zone 1, 7, and total ranged from 0.696 to 0.785 in the present study, we proposed that those nomograms had relative strong discrimination [22]. Our models also demonstrated reasonable calibration, as shown in Figs. 4, 5, and 6.

We found that the most highly influential factors for the postoperative periprosthetic bone loss were primary diagnosis and BMD in the corresponding Gruen zones at the baseline, which was insistent with previous studies. There was larger periprosthetic BMD decreases following THA for femoral neck fracture than for osteoarthritis [11]. As we discussed in our previous study [14], the trabecular bone of proximal femur became granular shaped and was located mostly in the interface between the implant and host bone after implantation of the femoral prosthesis. Similar to autogenous cancellous bone grafting, the trabecular bone would be eliminated before the new bone formation, which we supposed to be a reasonable explanation [31]. As for other selected variables in the nomograms, previous study demonstrated that younger patients have more postoperative daily living activities and corresponding accelerated periprosthetic bone remolding [32]. Similarly, we also found that age was negatively related to the postoperative bone loss in Gruen zone 1, 7, and total zones. Consistent with previous studies [13, 33], preoperative hip BMD was found to be predictable of less postoperative periprosthetic bone loss in the present study. Similar to our results, the meta-analysis reported that patients using straight stems experienced less bone loss than those using anatomic designs at the 1 year time point [12]. Nevertheless, further studies with larger scale and specific stem design groupings are necessary to determine its’ clinical relevance, as cementless anatomic stems were reported to be with satisfied survival rate at 10 years (> 95%) [34].

Our study was subjected to some limitations. Firstly, patients enrolled in the present study were relatively young (63, (51, 67) years, presented as median (Q1, Q3)). Further evaluation is needed before the application of those nomograms on older (> 80 years.) or much younger patients (< 40 years). Secondly, although the sample size of the present study has met the requirement of the statistics, we admitted that a large-scale sample is needed for building nomograms with higher discrimination and calibration. Besides, although the data was collected from a high-volume joint center that has a complex patient population, selection bias still existed due to the retrospective, single-center design. Lastly, external validation of the predictive model was not involved in the present study. As the sample size was relative small, we did not divide the data into training and test group in order to ensure adequate statistical power. Although the efficiency and accuracy of internal validation for nomogram has been proven in previous study [21, 35], we fully admit that external validation is necessary before generalized acceptance of these nomograms.

## Conclusion

To the best of our knowledge, our study represented the first time to use the nomograms in estimating the risk of postoperative periprosthetic bone loss with adequate predictive discrimination and calibration. We believed that those tools would help surgeons to identify high-risk patients who may benefit from anti-bone-resorptive treatment in the early postoperative period. Such prediction model could also provide patients with reasonable expectations following THA, which may improve satisfaction and patient compliance.

## Data Availability

The datasets used and/or analyzed during the current study are available from the corresponding author on reasonable request.

## Abbreviations

THA: Total hip arthroplasty
BMI: Body mass index
BMD: Bone mineral density
DEXA: Dual-energy X-ray absorptiometry
LSC: Least significant changes
ROI: Region of interest

## References

[1] Pincus D, Jenkinson R, Paterson M, Leroux T, Ravi B (2020). Association between surgical approach and major surgical complications in patients undergoing total hip arthroplasty. JAMA.

[2] Goodman SB, Gallo J (2019). Periprosthetic osteolysis: mechanisms, prevention and treatment. J Clin Med.

[3] Nam D, Barrack RL, Clohisy JC, Nunley RM (2016). Proximal femur bone density decreases up to 5 years after total hip arthroplasty in young, active patients. J Arthroplast.

[4] Tapaninen T, Kröger H, Venesmaa P (2015). Periprosthetic BMD after cemented and uncemented total hip arthroplasty: a 10-year follow-up study. J Orthop Sci.

[5] Nyström A, Kiritopoulos D, Ullmark G, Sörensen J, Petrén-Mallmin M, Milbrink J (2020). Denosumab prevents early periprosthetic bone loss after uncemented total hip arthroplasty: results from a randomized placebo-controlled clinical trial. J Bone Miner Res.

[6] Suzuki T, Sukezaki F, Shibuki T, Toyoshima Y, Nagai T, Inagaki K (2018). Teriparatide administration increases periprosthetic bone mineral density after total knee arthroplasty: a prospective study. J Arthroplast.

[7] Haugeberg G, Svenningsen S, Ugland SH, Berg ØH, Hugo Pripp A, Ugland TO (2018). Less periprosthetic bone loss following the anterolateral approach to the hip compared with the direct lateral approach. Acta Orthop.

[8] Wu X-D, Tian M, He Y, Chen H, Chen Y, Mishra R (2019). Short to midterm follow-up of periprosthetic bone mineral density after total hip arthroplasty with the ribbed anatomic stem. Biomed Res Int.

[9] Katz JN, Wright EA, Wright J, Malchau H, Mahomed NN, Stedman M (2012). Twelve-year risk of revision after primary total hip replacement in the U.S. Medicare population. J Bone Joint Surg Am.

[10] Goodnough LH, Finlay AK, Huddleston JI, Goodman SB, Maloney WJ, Amanatullah DF (2018). Obesity is independently associated with early aseptic loosening in primary total hip arthroplasty. J Arthroplast.

[11] Mann T, Eisler T, Bodén H, Muren O, Stark A, Salemyr M (2015). Larger femoral periprosthetic bone mineral density decrease following total hip arthroplasty for femoral neck fracture than for osteoarthritis: a prospective, observational cohort study. J Orthop Res.

[12] Knutsen AR, Lau N, Longjohn DB, Ebramzadeh E, Sangiorgio SN (2017). Periprosthetic femoral bone loss in total hip arthroplasty: systematic analysis of the effect of stem design. Hip Int.

[13] Fu G-T, Lin L-J, Sheng P-Y, Li C-C, Zhang J-X, Shen J (2019). Efficiency of zoledronic acid in inhibiting accelerated periprosthetic bone loss after cementless total hip arthroplasty in osteoporotic patients: a prospective, cohort study. Orthop Surg.

[14] Fu G, Ma Y, Liao J, Xue Y, Li M, Li Q (2020). High periprosthetic bone mineral density measured in immediate postoperative period may not guarantee less periprosthetic bone loss in the proximal femur after cementless total hip arthroplasty – a retrospective study. Arthroplasty.

[15] Mesko NW, Bachmann KR, Kovacevic D, LoGrasso ME, O'Rourke C, Froimson MI (2014). Thirty-day readmission following total hip and knee arthroplasty - a preliminary single institution predictive model. J Arthroplast.

[16] Goltz DE, Ryan SP, Hopkins TJ, Howell CB, Attarian DE, Bolognesi MP (2019). A novel risk calculator predicts 90-day readmission following total joint arthroplasty. J Bone Joint Surg Am.

[17] Wuerz TH, Kent DM, Malchau H, Rubash HE (2014). A nomogram to predict major complications after hip and knee arthroplasty. J Arthroplast.

[18] Collins GS, Reitsma JB, Altman DG, Moons KGM (2015). Transparent reporting of a multivariable prediction model for individual prognosis or diagnosis (TRIPOD): the TRIPOD statement. BMJ.

[19] Gruen TA, McNeice GM, Amstutz HC (1979). “Modes of failure” of cemented stem-type femoral components: a radiographic analysis of loosening. Clin Orthop Relat Res.

[20] Moons KGM, Altman DG, Reitsma JB, Ioannidis JPA, Macaskill P, Steyerberg EW (2015). Transparent reporting of a multivariable prediction model for individual prognosis or diagnosis (TRIPOD): explanation and elaboration. Ann Intern Med.

[21] Lubelski D, Alentado V, Nowacki AS, Shriver M, Abdullah KG, Steinmetz MP (2018). Preoperative nomograms predict patient-specific cervical spine surgery clinical and quality of life outcomes. Neurosurgery.

[22] Balachandran VP, Gonen M, Smith JJ, DeMatteo RP (2015). Nomograms in oncology: more than meets the eye. Lancet Oncol.

[23] Inaba Y, Kobayashi N, Oba M, Ike H, Kubota S, Saito T (2016). Difference in postoperative periprosthetic bone mineral density changes between 3 major designs of uncemented stems: a 3-year follow-up study. J Arthroplast.

[24] Khanuja HS, Vakil JJ, Goddard MS, Mont MA (2011). Cementless femoral fixation in total hip arthroplasty. J Bone Joint Surg Am.

[25] Messina C, Usuelli FG, Maccario C, Di Silvestri CA, Gitto S, Cortese MC (2019). Precision of bone mineral density measurements around total ankle replacement using dual energy X-ray absorptiometry. J Clin Densitom.

[26] Zhou W, Liu Y, Guo X, Yang H, Xu Y, Geng D (2019). Effects of zoledronic acid on bone mineral density around prostheses and bone metabolism markers after primary total hip arthroplasty in females with postmenopausal osteoporosis. Osteoporos Int.

[27] Aro E, Moritz N, Mattila K, Aro HT (2018). A long-lasting bisphosphonate partially protects periprosthetic bone, but does not enhance initial stability of uncemented femoral stems: a randomized placebo-controlled trial of women undergoing total hip arthroplasty. J Biomech.

[28] Huang T-W, Wang C-J, Shih H-N, Chang Y, Huang K-C, Peng K-T (2017). Bone turnover and periprosthetic bone loss after cementless total hip arthroplasty can be restored by zoledronic acid: a prospective, randomized, open-label, controlled trial. BMC Musculoskelet Disord.

[29] Yukizawa Y, Inaba Y, Kobayashi N, Choe H, Kubota S, Saito T (2017). Efficacy of alendronate for the prevention of bone loss in calcar region following total hip arthroplasty. J Arthroplast.

[30] Ryan SP, Goltz DE, Howell CB, Jiranek WA, Attarian DE, Bolognesi MP (2019). Predicting costs exceeding bundled payment targets for total joint arthroplasty. J Arthroplast.

[31] Roberts TT, Rosenbaum AJ (2012). Bone grafts, bone substitutes and orthobiologics: the bridge between basic science and clinical advancements in fracture healing. Organogenesis.

[32] Hayashi S, Hashimoto S, Kanzaki N, Kuroda R, Kurosaka M (2016). Daily activity and initial bone mineral density are associated with periprosthetic bone mineral density after total hip arthroplasty. Hip Int.

[33] van der Wal BCH, Rahmy A, Grimm B, Heyligers I, Tonino A (2008). Preoperative bone quality as a factor in dual-energy X-ray absorptiometry analysis comparing bone remodelling between two implant types. Int Orthop.

[34] Harada Y, Mitsuhashi S, Suzuki C, Yamashita K, Watanabe H, Akita T (2007). Anatomically designed prosthesis without cement for the treatment of osteoarthritis due to developmental dysplasia of the hip: 6- to 13-year follow-up study. J Orthop Sci.

[35] Gronbeck CJ, Cote MP, Halawi MJ (2019). Predicting inpatient status after total hip arthroplasty in medicare-aged patients. J Arthroplast.

